# Establishment, maintenance and application effect analysis of the prescription pre-review system in a tertiary hospital in China

**DOI:** 10.1186/s12913-025-12901-8

**Published:** 2025-07-01

**Authors:** Li Zhou, Xi Gu, Feilong Tan, Yanhua Li, Wenjing Duanmu, Hongyi Che, Fan Yang, Wenjie Yin

**Affiliations:** 1 Department of Pharmacy, The Affiliated Yan’an Hospital of Kunming Medical University, Kunming, China; 2 Joint Surgery, General Hospital of Xizang Military Command, Lhasa, China

**Keywords:** Prescription pre-review, Rules maintenance, Warning levels, Pharmacist intervention, Rational drug use

## Abstract

**Background:**

Implementing a full-sample prescription review system has become imperative for hospitals to address the limitations of post-review practices and ensure patient medication safety. In this study, we investigate the preliminary application impact of the prescription pre-review system (PPRS)and its rules maintenance in our hospital, while also evaluating the pharmaceutical service value resulting from the implementation of prescription pre-review.

**Methods:**

We incorporated levels of prescribing warnings into the PPRS, while establishing and maintaining a database of appropriate medication practices. In addition, we evaluated the rationality of intravenous infusion prescriptions and key monitored drug prescriptions before and after the implementation of PPRS, and briefly evaluated the irrational prescriptions.

**Results:**

A comprehensive range of 7 warning levels was established, reflective of the degree of prescription irrationality. Besides, a total of 3015 user rules were created to regulate medication behavior. The number and proportion of unreasonable prescriptions during January to March 2023 demonstrated a noteworthy reduction when compared to the corresponding period in 2022. Moreover, the continuous evaluation of systematic alert prescriptions during January to March 2023 indicated a remarkable monthly decline.

**Conclusion:**

The introduction of the PPRS has ushered in a paradigm shift in the evaluation of prescription rationality. This transformative system has played a pivotal role in standardizing doctor’s prescribing patterns, thereby promoting the rational utilization of drugs in clinical practice. By ensuring patient safety, elevating the quality of care, and enhancing the value of pharmacists, the PPRS has emerged as a catalyst for positive change in the healthcare setting.

**Supplementary Information:**

The online version contains supplementary material available at 10.1186/s12913-025-12901-8.

## Background

Prescription review is a critical aspect of healthcare delivery that aims to assess the appropriateness of prescriptions and drugs within the entire patient population [1]. Establishing standardized procedures is essential to guide hospital clinicians, pharmacists, and medical management professionals in their search for robust evidence that confirms the rationality of prescriptions [2–4]. This ensures that patients receive drugs safely, effectively, economically, and reasonably [5, 6].

The Ministry of Health of the People’s Republic of China recognized the importance of standardizing the review of hospital prescriptions and improving prescription quality, rational drug use, and medical safety. In 2010, they formulated the “Management Standard of Hospital Prescription Comment(Trial) [7]” in accordance with relevant laws and regulations, including the “Drug Administration Law“ [8] and “Prescription Administration Measures“ [9]. This standard outlines the prescription review process, which involves evaluating the standardization of prescription writing and the suitability of clinical drug use. To effectively implement prescription review, hospitals must focus on strengthening prescription quality and drug clinical application management, which involves standardizing doctors’ prescription behavior and implementing relevant regulations such as prescription review, drug distribution, check, and medication explanation. Such measures ensure that patients receive optimal care, while also contributing to overall healthcare delivery improvements.

Building upon these efforts, the Pharmaceutical Affairs Committee of the Chinese Hospital Association introduced the “Guideline for the evaluation of prescription appropriateness” to further standardize rational drug use [10]. Nonetheless, the current challenge lies in the large volume of hospital prescriptions, the extensive range of content and directions requiring review, and the insufficient number of reviewers. As a result, there are limitations and delays in post-reviewing prescriptions [11, 12]. Prescription post-review is a process where pharmacists review the prescriptions for reasonableness, safety, and compliance before the patient actually receives the medication after the physician has written the prescription and the patient has completed payment. This process in our hospital is reviewers manually review and approve the prescriptions, which has problems such as time lag, high labor cost, information asymmetry, incomplete review, and increased patient waiting time. Consequently, implementing a full-sample prescription pre-review in hospitals has become crucial to ensuring patient medication safety. Recognizing the importance of this issue, the National Health Commission (NHC) issued the “Notice on the Issuance of prescription review Standards for Medical Institutions” on July 10, 2018 [13]. This notice clearly mandates that all prescriptions must receive approval before entering the pricing, charging, and dispensing process. The review and approval process is carried out by qualified pharmacists of the Hospital, and the process is as follows: (a) The pharmacist receives the prescription to be reviewed, and reviews the prescription for legality, standardization, and appropriateness. (B) If the audit is determined to be a reasonable prescription, the pharmacist in the paper prescription handwritten signature (or stamped with a special seal), electronic signature on the electronic prescription, the prescription signed by the pharmacist to enter the charges and dispensing links. (c) If the prescription is judged to be unreasonable after the audit, the pharmacist will be responsible for contacting the prescribing physician to ask him/her to confirm or reissue the prescription and enter the prescription audit process again. Prescription charges or dispensing should not occur without prior approval. Studies based on this standard have demonstrated that compared to post-review methods, pre-review and intervention in prescriptions are more immediate, effective, and comprehensive. They better address the safety, effectiveness, and economy needs of most patients for drugs, thereby reducing the occurrence of adverse drug events, medical risks, and medication error disputes [14–16].

To enhance the rationality of drug use and ensure the safety of patient medication, our hospital officially launched the prescription pre-review system (PPRS) in April 2022. Through this system, we aim to transition from post-review to full sample pre-review of prescription appropriateness. This article comprehensively discusses the establishment and application of the PPRS in our hospital, analyzing the improvement in the rationality of key prescriptions. Additionally, it explores the impact of PPRS on the quality of pharmaceutical care.

## Methods

### Sources of PPRS

The PPRS utilized in our hospital is called the JINPE intelligent assistant decision-making system for safe medication, which was procured from Xiamen Jingpei Software Engineering Co., LTD (Fujian Province, China). This system incorporates principles and technology derived from computer database structure, enabling prescription pre-review based on the fundamental characteristics of clinical rational drug utilization and the specific demands of rational drug management in medical institutions, with the drug instructions as the authoritative prescription review standard.

### Establishment of prescription reviewing pharmacists

The Pharmacy Department within our hospital has established a prescription review team, which comprises two junior pharmacists and three intermediate pharmacists. Junior pharmacists are hospital pharmacy staff who have graduated from a pharmacy program with a degree in pharmacy and have passed the National Qualification Examination for Health Professions and Technology to obtain a junior pharmacist certificate. Intermediate pharmacists are pharmacy personnel who have accumulated more clinical experience and professional knowledge through further study and practice on the basis of junior pharmacists and have been awarded the Intermediate Pharmacist Certificate by the state. On this basis, all team members have successfully completed the National Information Pharmacist Training jointly organized by the Pharmaceutical Information Committee of the Chinese Pharmaceutical Society and the Guangdong Pharmaceutical Society, focusing on pharmacy information services, prescription review, and medication therapy management. And received the Information Pharmacist Certificate issued by them. Information pharmacists are pharmacists primarily engaged in the management and application of pharmacy information, and their work focuses on supporting clinical decision-making, optimizing drug therapy, enhancing patient safety and improving medical outcomes through effective information management, analysis and delivery. Meanwhile, the team members passed the prescription review pharmacist training organized by the China Association for Pharmaceutical Education and obtained the prescription review qualification certificate issued by the association. The team operates on a strict 24-hour shift schedule (excluding legal holidays), and through the real-time e-prescribing review interface, they collaborate and rotate the review of physician-submitted prescriptions, orders, and reasons for interventions, and provide electronic prescription comments on the review page in a timely manner for erroneous prescriptions. The results of the review and comments are sent to the physician’s prescribing electronic system page in a timely manner, and the physician makes corrections or appeals accordingly. Feedback on these reviews is promptly provided to the doctors within 60 s, with a specific focus on contacting and reminding them to address any incorrect prescriptions or medication contraindications that require attention.

Additionally, to address inevitable challenges associated with off-label drug usage, pharmacists are encouraged to enhance communication with clinicians. They inform clinicians to support off-label use applications by providing evidence-based medicine evidence and emergency plans. The evidence of which should be in accordance with the management guideline for the off-label use of medicine in China [17]. The application for off-label drug utilization is subject to comprehensive review by the pharmacy department, medical department, hospital pharmaceutical administration committee, and hospital ethics committee prior to implementation [18]. Once approved, the prescription review team incorporates the relevant content into the drug rules of the PPRS, thereby eliminating system warnings related to the associated off-label usage. A number of medicines are now approved for off-label use and the rules have been changed accordingly. For example, fluticasone propionate suspension for nebulized inhalation has been increased to adults and adolescents over 16 years of age; methotrexate injection is injected directly into the sac of pregnancy in the fallopian tube; olanzapine tablets are used for antiemetic purposes in patients undergoing chemotherapy; and the route of administration of gemcitabine injections has been increased to include bladder perfusion, among others.

### System maintenance and problem communication

The maintenance of the PPRS is a collaborative effort involving various professionals, forming an efficient closed-loop maintenance management system. This includes system engineers, hospital information system (HIS) engineers, information department engineers, prescription review pharmacists, clinical pharmacists, and dispensing pharmacists. That is, based on a clearly established audit process, the pharmacists conduct real-time monitoring and feedback of medication information, as well as enhanced communication with physicians, active patient education, and regular medication training. During this process, the medication rules are adjusted in a timely manner according to clinical needs and evidence of medication use. During this period, engineers are responsible for providing IT support in all aspects. This collaborative effort between them ensures the effective maintenance of the PPRS and the provision of quality pharmaceutical services within our hospital.

### Rule base construction and maintenance

The medication rule base is a database inside the PPRS that contains various review rules and criteria. These rules and criteria are used to guide the pharmacist or review system in making judgments and decisions when reviewing prescriptions. The main responsibility for constructing the rule base lies with system engineers and prescription review pharmacists, who work together in a cooperative manner. The maintenance process, on the other hand, is led by prescription review pharmacists, with support from engineers. The maintenance of the rule base involves the upkeep of drug instructions, drug rule matching (the process by which the system automatically compares and evaluates prescriptions written by physicians for compliance with predefined rules and criteria for drug use), and large rules (a core set of universally applicable audit rules. Usually generalized rule restrictions for a few or certain types of drugs). The maintenance activities cover the entire operation process, both before and after the system is launched. Generally, the maintenance of drug instructions primarily entails ensuring the accuracy of manufacturers, specifications, and update dates of the drug instructions used in our hospital. Matching and maintaining drug rules is primarily based on the addition or update of instructions and the management of warning level changes during the prescription review process. This matching and maintenance process is carried out throughout the entire prescription review process. Unlike the maintenance of individual drug rules, the maintenance of large rules extends to all drugs or a specific class of drugs in the hospital. Once a large rule is successfully constructed, it imposes restrictions on all drugs within the corresponding class.

### Data preparation and statistical analysis

A retrospective analysis and comparison were conducted using a sample of intravenous drug prescriptions and key monitored drug prescriptions from January to March 2022 before the implementation of the PPRS, and from January to March 2023 after the implementation of the PPRS. As planned, we took a simple random sampling method to extract prescriptions from January-March 2022 and the same period in 2023 for comparison. In order to fully capture and have a comprehensive picture of prescription rationalization, we took all appropriate prescriptions for the month to determine the sample size. Prescriptions in which the prescription was clearly executed with complete patient information, diagnostic information, medication information, and cost records were included. Repeated prescriptions, prescriptions deleted by the doctor but still recorded in the system, and prescriptions with incomplete patient information and medication information were excluded. The sample consisted of 201,112 intravenous drug prescriptions and 10,005 key monitored drug prescriptions before the PPRS, and 162,899 intravenous drug prescriptions and 44,674 key monitored drug prescriptions after the PPRS. Furthermore, the study also assessed the types and proportion of unreasonable prescriptions and the intervention of pharmacists from January to March 2023 after the PPRS was launched. The original data were obtained from the Prescription Lookup section of PPRS, and all studies were retrospective in nature.

To analyze the data, Statistical Package for the Social Sciences (SPSS) 22.0 statistical software was used for data calculation and processing. Since the data in this study are binary qualitative data, they are all described in the form of frequency and percentage, and expressed as (n, %), only Chi-square test is used for comparison. A p-value less than 0.05 was considered statistically significant.

## Findings and results

### Construction and implementation of PPRS

**Fig. 1:**
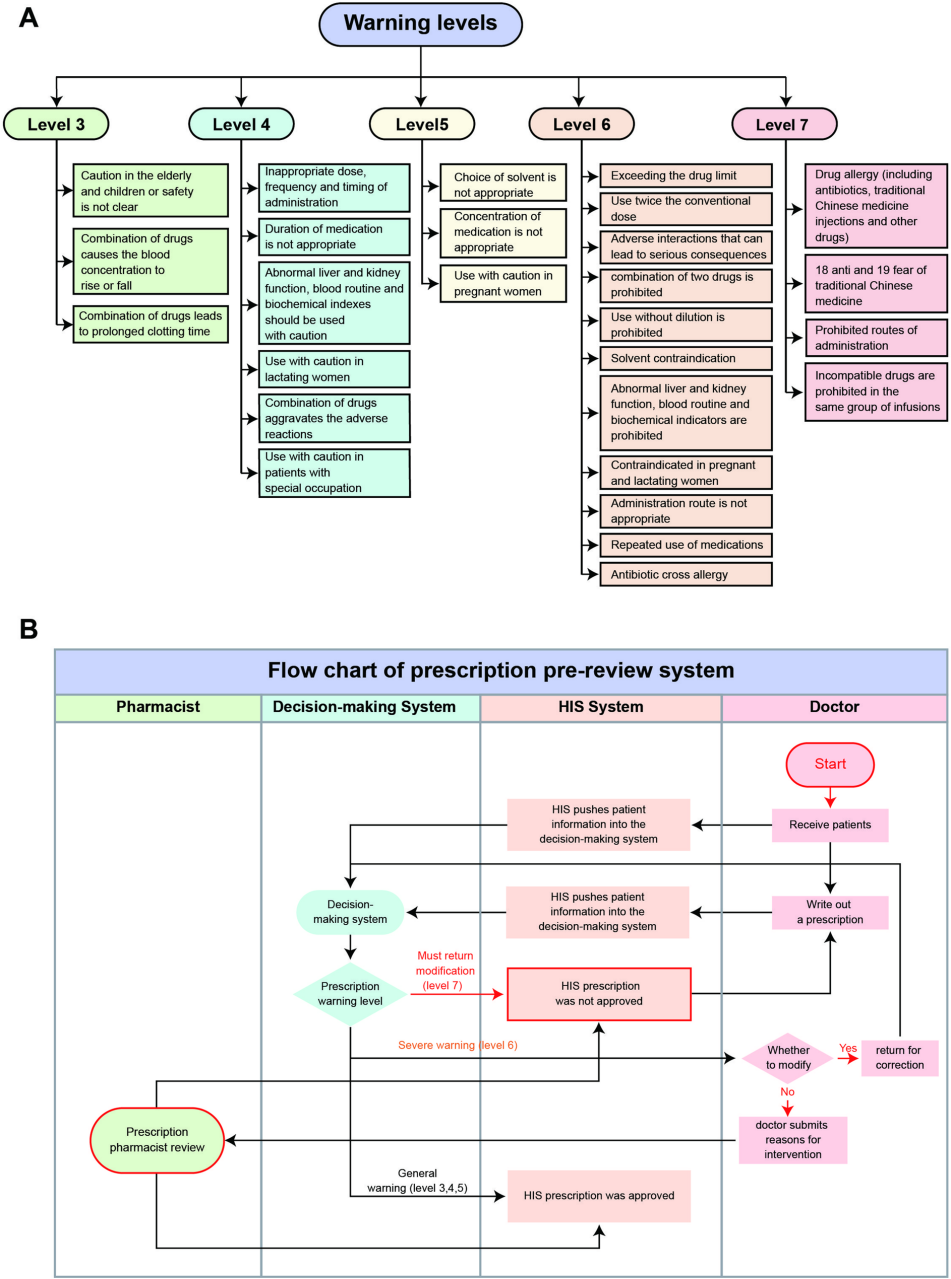
**A** Work flow chart of PPRS, **B** Warning levels and contents of the hospital

The PPRS in the hospital is integrated with the HIS through an interface, allowing for the transmission of basic data. The rationality of drug use is reviewed and evaluated in strict accordance with the Prescription Review Standards for Medical Institutions. A flowchart depicting the process is shown in Fig. 1A. In this process, the HIS pushes prescription or medical order information to the PPRS. The PPRS then generates warnings based on the level of the problem prescription. Warnings of levels 4–7 are displayed in the doctor’s workstation, while warnings of level 3 are only displayed in the background. 

Based on the warning level and modification suggestion, the doctor has several options. For warning levels 4–5, the doctor can choose to save the prescription or return it for modification based on the warning suggestion. For level 6 warnings, the doctor can return the prescription for modification or fill in the reason for use and submit it to the reviewing pharmacist for review. If the review by the pharmacist is passed, the prescription can be saved directly. If the review fails, the prescription can only be returned for modification. Prescriptions for level 7 warnings must always be returned for modification. However, if there are special reasons for prescribing the medication at level 7, the doctor can explain the reason to the reviewing pharmacist. In such cases, the pharmacist can downgrade the warning level, allowing the prescription to be re-prescribed.

The warning levels in the PPRS range from 1 to 10, with levels 3–7 being commonly used. The hospital has standardized the warning levels and content based on the actual situation, as shown in Fig. 1B. Simply put, level 3 warnings include caution for the use of medications in elderly and pediatric patients or when safety is not yet established, as well as cases where co-administration of drugs may alter blood drug concentrations or prolong clotting time. Level 4 warnings include inappropriate dosage, frequency, or timing of administration, abnormal biochemical indicators, caution for lactating women, and caution for patients in specific professions. Level 5 warnings involve inappropriate choice of solvents or drug concentrations, and caution for pregnant women. Level 6 warnings include excessive drug dosages, doses exceeding conventional limits by 2 times, adverse interactions that can lead to severe consequences, prohibition based on abnormal biochemical indicators, and repeated medication. Level 7 warnings encompass drug allergies, prohibited routes of administration, and the eighteen contraindications and nineteen precautions of traditional Chinese medicine.

### Creation and maintenance of user rules

**Fig. 2:**
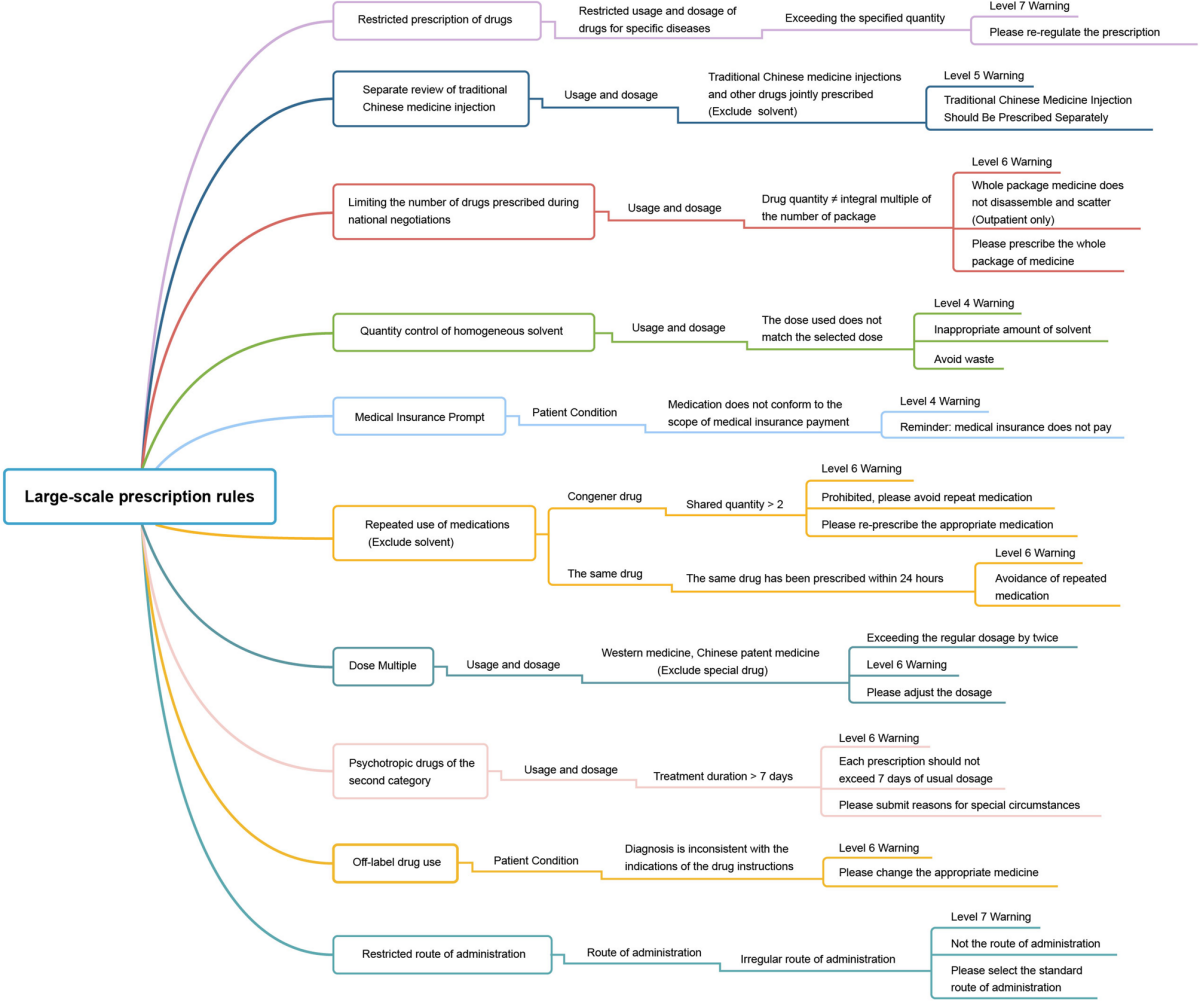
Creation and maintenance of large-scale prescription rules

Since the implementation of the PPRS, a total of 3015 user rules were created. These rules were categorized into different types, including 2335 rules for drug instructions (including most of the drug instructions and rules in our hospital), 670 rules for modifying single drug prescriptions (including the modification or update of the rules of usage, dosage, route of administration, patient condition, biochemical index, etc.), and 10 rules for the creation and modification of large-scale prescription rules (brief information is shown in Fig. 2). The maintenance and utilization of these user rules not only adhered to the national medication standards but also catered to the specific medication needs of the hospital.

The implementation of these user rules has significantly reduced the rate of prescription errors. This, in turn, enhanced patient safety during medication. The PPRS has been well received by both doctors and patients. Furthermore, Efforts are continually being made to improve and update our system rules and user rules. The objective is to further standardize medication practices and provide better guidance for the well-being and health of patients.

### The effect of PPRS on prescription intervention and prescription qualification rate

#### Comparison of irrational prescription of intravenous drugs

The administration of intravenous drugs requires utmost caution as any misuse can lead to severe adverse consequences [3, 19–21]. Our hospital recognizes the significance of this and places great emphasis on the proper use of intravenous drugs. Tables 1 and 2 display the reduction in errors in intravenous drug prescriptions during the months of January to March in 2023 compared to the same period in 2022. Compared to the same period in 2022, there was a decrease in total intravenous drug prescriptions in 2023, averaging about 10,000 fewer per month. Additionally, the number of irrational prescriptions also significantly decreased. The results of the chi-square test indicated that the difference in error rates is statistically significant. This fully addressed the call to increase the standardized use of intravenous fluids in hospitalized patients, as outlined in the “National Healthcare Quality and Safety Improvement Goals for 2023” issued by the General Office of the NHC [22].

**Table 1:** Comparison of irrational intravenous drug prescriptions requiring manual intervention by pharmacists before and after the use of PPRS in different months of the same year

Year	Month	Number of unqualified prescriptions	Number of qualified prescriptions	X^2^	*P*
2022	January	67 (0.1)	67,861 (99.9)	5.646	0.059
	February	39 (0.1)	58,749 (99.9)		
	March	77 (0.1)	74,319 (99.9)		
2023	January	6 (0)	54,596 (100.0)	0.555	0.758
	February	5 (0)	52,341 (100.0)		
	March	8 (0)	55,943 (100.0)		

**Table 2:** Comparison of irrational intravenous drug prescriptions requiring manual intervention by pharmacists in the same month before and after the use of PPRS

Month	Year	Number of unqualified prescriptions	Number of qualified prescriptions	X^2^	*P*
January	2022	67 (0.1)	67,861 (99.9)	48.014	< 0.001
	2023	6 (0)	54,596 (100.0)		
February	2022	39 (0.1)	58,749 (99.9)	22.564	< 0.001
	2023	5 (0)	52,341 (100.0)		
March	2022	77 (0.1)	74,319 (99.9)	38.992	< 0.001
	2023	8 (0)	55,943 (100.0)		

Since the implementation of the PPRS, it effectively identified and rectified prescription errors related to intravenous drugs, which included timely alerts and corrections for incorrect solvents or diluents, inappropriate infusion fluid volume, and incompatibilities. These interventions successfully standardized doctors’ prescribing practices and significantly reduced prescription errors. Moreover, the number of unjustified intravenous drug prescriptions and those requiring pharmacist review markedly decreased. This reduction ensured the safety of patients receiving infusion treatments and underscored our commitment to medication safety.

#### Comparison of irrational prescription of key monitored drugs

Enhancing the review and evaluation of prescriptions for key monitored drugs holds immense importance in promoting rational drug usage and safeguarding the rights and interests of individuals’ health [23]. The General Office of the NHC has emphasized that the list of rational drug use under key monitoring should encompass chemical and biological products that exhibit significant irrationalities in clinical utilization, excessive expenditure, and substantial impact on the rationality of drug use. The focus is directed towards adjuvant drugs, antineoplastic drugs, antimicrobial drugs, proton pump inhibitors, glucocorticoids, parenteral nutrition drugs, among others [24].

To reinforce comprehensive management throughout the entire process of clinical drug application and further standardize doctors’ prescription practices, it is essential to conduct prescription reviews for all drugs included in the list. Additionally, it is necessary to strengthen the publicity, feedback, and utilization of the outcomes of prescription reviews [25–28]. Our hospital developed a list of key monitored drugs specific to our institution, combining the national and provincial lists with the actual drug utilization patterns. Currently, this list comprised 70 drugs (Supp. Table 1), all of which undergo stringent prescription reviews. The implementation and usage of the PPRS significantly standardized the correct utilization of these drugs. As illustrated in Tables 3 and 4, the total number of prescriptions for the key monitored drugs increased in 2023 compared to the same period in 2022. However, there was no significant increase in irrational prescriptions. Moreover, the prescription qualification rate considerably improved compared to the previous period, with a statistically significant difference observed. We aspire to continuously enhance the PPRS and optimize user guidelines to better oversee the usage of key monitored drugs and advance the rational use of medications.

**Table 3:** Comparison of prescription rationality of key monitored drugs in different months of the same year before and after PPRS implementation

Year	Month	Number of unqualified prescriptions	Number of qualified prescriptions	X^2^	*P*
2022	January	952 (23.4)	3123 (76.6)a	8.480	0.014
	February	894 (23.9)	2850 (76.1)a		
	March	581 (26.6)	1605 (73.4)b		
2023	January	959 (7.3)	12,248 (92.7)a	179.487	< 0.001
	February	470 (3.6)	12,702 (96.4)b		
	March	950 (5.2)	17,345 (94.8)c		

The results of the groups marked “a”,” b” and “c” were compared, and the difference was statistically significant

**Table 4:** Comparison of prescription rationality of key monitored drugs in the same month before and after PPRS implementation

Month	Year	Number of unqualified prescriptions	Number of qualified prescriptions	X^2^	*P*
January	2022	952 (23.4)	3123 (76.6)	820.825	< 0.001
	2023	959 (7.3)	12,248 (92.7)		
February	2022	894 (23.9)	2850 (76.1)	1622.205	< 0.001
	2023	470 (3.6)	12,702 (96.4)		
March	2022	581 (26.6)	1605 (73.4)	1291.19	< 0.001
	2023	950 (5.2)	17,345 (94.8)		

#### Summary of the unreasonable types of pre-reviewed prescriptions

Table 5 below shows the main unreasonable types of outpatient prescriptions (only outpatients are counted here) found by prescription pre-review from January to March 2023. According to the data obtained from the prescription pre-review monitored by the PPRS, the main types of irrational prescriptions identified in our hospital during the period from January to March were primarily related to drug interactions, inappropriate usage and dosage, incompatibility, and unsuitable solvent selection. These issues mainly triggered level 3–5 warnings in the system, which served as reminders to doctors for proper prescribing behavior. It did not require pharmacist intervention and review, but rather sought to influence doctors’ prescription practices. The proportion of warning prescriptions displayed a downward trend, indicating that the warnings at different levels within the PPRS gradually influenced and standardized doctors’ prescribing behavior. In future prescription reviews, pharmacists will strengthen the supervision of irrational drug use through warnings and remind doctors to standardize their prescriptions. This will contribute to an increase in the prescription qualification rate and the rationality of drug usage, thus ensuring the safety of patients’ medication.

**Table 5:** Summary of irrational prescription alerts from the PPRS in January-March 2023

Time	Number of prescriptions					Total number of prescriptions	Proportion of warning prescriptions
	Drug interactions	Inappropriate usage and dosage	Incompatibility	Unsuitable solvent selection	Others		
January	8663	2150	332	67	3115	61,053	23.47%
February	9486	1858	234	67	3315	69,927	21.39%
March	11,518	3235	575	62	4589	100,420	19.89%

## Discussion

### Promoting rational drug use and ensuring drug safety

The concept of drug-related burden, which includes both physical and economic burdens, has gained increasing attention due to the prevalence of polypharmacy. Polypharmacy can impact patients’ medication behavior, leading to nonadherence and medication errors [29–31]. It is crucial to focus on patients’ acceptance of rational drug use guidance and implement measures to enhance medication safety and application guidance systems. By doing so, the value of safe drug use can be fully realized and patients can have more trust and confidence in the prescribed medications [32–35]. This will ultimately improve medication compliance and enhance recognition, satisfaction, and support for prescription pre-review, leading to better patient service and improved medication safety [28, 36–38]. In comparison to previous post-prescription review practices, the prescription pre-review process achieves a closed-loop audit management approach that includes pre-review, in-process supervision, and post-evaluation. Furthermore, the review scope encompasses all prescriptions and medical orders from various clinical departments. The results of our PPRS practice prove that the real-time prescription review efficiently identifies irrational prescriptions, timely standardizes doctors’ prescription behavior, and improves the quality of prescriptions. This strengthens patient drug safety measures and promotes rational drug use in clinical settings.

### Improving the quality of healthcare and the value of pharmacists

The implementation and utilization of the PPRS aim to utilize information effectively and elevate basic pharmaceutical services to a higher level of pharmaceutical professional services. This also enhances the value of pharmacists. Up to now, the transition from manual prescription review to an electronic prescription review system-led review has been successful, as has the shift from post-review to pre-review. The volume and accuracy of comments have increased, and the work efficiency of pharmacists has significantly improved. During the prescription pre-review process, pharmacists gain a comprehensive understanding of commonly used drugs, their characteristics, and contraindications, as well as key drugs. They also have more time to study personalized drug use and address controversial prescription review issues by consulting authoritative data (pharmacopoeias, medical textbooks, guidelines, etc.). This promotes evidence-based prescribing and the incorporation of clinical guidelines into clinical practice [30, 39, 40]. Through this process, prescription reviewing pharmacists can augment their pharmaceutical knowledge and skills while enhancing their ability to review prescriptions. This, in turn, standardizes the rational prescribing practices of doctors, earns clinical recognition, and elevates the value of pharmacists. 

### Improving the economics of drug therapy

From a pharmacoeconomic perspective, the effective operation and development of PPRS reduces the cost of drug use. This, in turn, reduces the cost of healthcare, alleviates the financial burden on patients, and enhances the credibility and sustainability of healthcare organizations. The misuse of drugs can result in wastage and increased medical expenses [41–43]. However, prescription pre-review can help identify and correct irrational drug use, such as overuse and abuse, in a timely manner. This can prevent drug wastage, improve drug efficiency, and reduce costs. Moreover, by creating and maintaining rules in PPRS, it is possible to select drugs rationally and optimize their use based on the patient’s condition and drug characteristics. This approach not only enhances the therapeutic effect of drugs but also reduces the cost of drug use. The operational efficiency of healthcare organizations directly impacts their economic benefits [44, 45]. The use of PPRS can improve efficiency and reduce the workload of pharmacist audits, thus enhancing economic benefits. Furthermore, during prescription pre-review, drug usage issues can be identified, providing a reference for drug development and innovation. This can lead to the creation of new drugs that improve therapeutic effectiveness and reduce medication costs. In summary, the implementation of the PPRS application has had a positive impact on pharmacoeconomics.

### Deficiencies and improvement of PPRS

Despite its implementation, the PPRS still faces several challenges within the hospital setting. One of the issues is the lack of timely maintenance and updates for the drug instructions in the system. Additionally, the instructions available do not cover all the drugs used within the hospital. Moreover, some system rules were directly copied from the instructions without proper updates or inclusion of all necessary information. As a result, some unreasonable medication conditions are not correctly flagged. As prescription reviewing pharmacists, we spend a significant amount of time checking the drug instructions within the system during the initial stages of its launch. When there is a need for maintenance, updates, or rule modifications, we have to provide the drug instructions to the system engineer for completion. During system usage, we also need to make changes to the content and level of corresponding rules based on guidelines, authoritative materials, and the specific needs of our hospital. However, if these changes are not promptly implemented and updated in the application, it leads to inappropriate prompts being shown to doctors, potentially resulting in error alerts. Furthermore, due to poor integration between the PPRS and the HIS, the PPRS cannot accurately retrieve certain information such as height, weight, liver function, kidney function, and other bio-indicators for some outpatients. This lack of accurate information makes it challenging to assess the impact of these biochemical indicators on medication and provide precise individualized guidance for patients’ medication. Hence, there is a need for continuous improvement in this area. Additionally, during the initial stages of implementing the system, discrepancies arose due to variances in the professional expertise of prescription reviewing pharmacists and the subjective nature of judgment in reviewing prescriptions. As a means to mitigate these subjective differences, the prescription review team organizes periodic discussions on prescription review matters. These discussions aim to formulate treatment measures, establish a rule base for prescription review, and standardize the passing and failing criteria for the review process. This approach ensures a more consistent and objective assessment of prescriptions.

Our study was conducted under the condition of a basically fixed healthcare team without any quality improvement plans. This eliminated result biases caused by staff changes and quality adjustments, ensuring the effectiveness and validity of the study. However, there are limitations to the widespread and effective use of the PPRS under the same criteria, due to potential factors such as variations in hospital size, patient numbers, inconsistent healthcare infrastructure, changes in medical quality control requirements, as well as uncontrollable factors like seasonal variations, epidemics, earthquakes, etc. The research results from a certain period of PPRS study are insufficient to fully demonstrate and support the absolute feasibility and wide applicability of creating and maintaining PPRS. Therefore, in future research, we will continue to track prescription review situations over a longer time period, pay continuous attention to the effective prompts of different warning levels in the PPRS system, mitigate potential risks, and evaluate the accuracy and sustainability of establishing and using the PPRS. Additionally, with appropriate support, we will collaborate with other comprehensive tertiary hospitals to further explore the contributions of the rational application of the PPRS in the fields of healthcare and medication safety.

## Conclusion

This study provides a brief introduction to the construction of prescription warning levels, the creation and maintenance of medication rules, and preliminary exploration of changes in prescription rationality before and after the implementation of the PPRS. In the future, further in-depth research will be conducted. Though the path may be challenging and long, progress will be made. The utilization of the PPRS system effectively harnesses the advantages of information-based pharmaceutical services, leading to a promotion of rational drug utilization in clinical settings. This, in turn, enhances patient medication safety, reduces the cost of medication, improves the quality of healthcare, and adds value to the role of pharmacists. The establishment and refinement of a rule base, along with enhancements to the review process and intervention system, facilitate better communication between pharmacists and doctors. This enables the exchange and sharing of effective medication experiences and guidance, ultimately improving pharmacists’ ability to analyze medication rationality based on evidence. These efforts lay the foundation for the further standardization of pharmaceutical services, thereby safeguarding patients’ lives and health.

## Supplementary Information

Supplementary Material 1.

## Data Availability

All data generated or analysed during this study are included in this published article.

## Abbreviations

PPRS: Prescription Pre-review System
NHC: National Health Commission
HIS: Hospital Information System
SPSS: Statistical Product and Service Solutions
