# Supplementary material for: Establishment, maintenance and application effect analysis of the prescription pre-review system in a tertiary hospital in China
Source: BMC Health Serv Res. 2025 Jul 1;25:853. doi: 10.1186/s12913-025-12901-8 (PMC12220340; doi:10.1186/s12913-025-12901-8)
Supplement: Supplementary file 1 — Supplementary Material 1. [file 12913_2025_12901_MOESM1_ESM.docx]

**Key Monitored Drugs List**

| **Drug Code** | **Drug Name** | **Specification** | **Manufacturer** |
| --- | --- | --- | --- |
| 1303011PW1 | Budesonide Nebulizer Suspension | 1mg | AstraZeneca Pty Ltd |
| 1303042XR1 | Nebulized Budesonide Suspension for Inhalation | 2ml:1mg | Sichuan Prat Pharmaceutical Co., Ltd. |
| 1303047XR1 | Budesonide Inhalation Aerosol | 20mg:0.1mg*200 doses/bottle | Lunan Betta Pharmaceutical Co., Ltd. |
| 1201026PJ1 | Piracetam Tablets | 0.4g*30 tablets | Jiangxi Yoyoo Pharmaceutical Co., Ltd. |
| 1201018JN1 | Oxyracetam Capsules | 0.4g*24 capsules | CSPC Ouyi Pharmaceutical Co., Ltd. |
| 1401055ZS0 | Aprepitant for Injection | 10mg | Livzon Pharmaceutical Group Inc. |
| 1099017ZS2 | Lobaplatin for Injection | 10mg | Hainan Chang'an International Pharmaceutical Co., Ltd. |
| 1000003ZJ1 | Camrelizumab for Injection | 200mg | Suzhou Shengdiya Biopharmaceutical Co., Ltd. |
| 1209039ZJ1 | Compound Brain Peptide and Ganglioside Lipid Injection | 2ml | Jilin Buchang Pharmaceutical Co., Ltd. |
| 0604010ZS1 | Multivitamin Injection (12) | 5ml/bottle | Shanxi Pude Pharmaceutical Co., Ltd. |
| 0604011ZS2 | Compound Vitamin B3 Injection (II) | Compound | Jilin Jinsheng Pharmaceutical Co., Ltd. |
| 1003019ZS1 | Arsenic Trioxide Injection | 10mg | Beijing Shuanglu Pharmaceutical Co., Ltd. |
| 1209036ZS2 | L-Alanyl-L-Glutamine for Injection | 10g | Hainan Lingkang Pharmaceutical Co., Ltd. |
| 1899005SY2 | Sodium Acetate Ringer's Injection | 500ml | Hubei Duorui Pharmaceutical Co., Ltd. |
| 0802014ZS1 | Hydrocortisone Injection | 10mg*2ml | Jiangxi Guoyao Co., Ltd. |
| 0802019ZS1 | Methylprednisolone Sodium Succinate for Injection | 0.5g | Pfizer Manufacturing Belgium NV |
| 0802037ZJ1 | Methylprednisolone Sodium Succinate for Injection | 40mg | Liaoning Haisco Pharmaceutical Co., Ltd. |
| 0802036ZJ1 | Dexamethasone Acetate Injection | 1ml:5mg | Chengdu TianTaiShan Pharmaceutical Co., Ltd. |
| 0401052ZS2 | Pentazocine Injection (Narcotics II) | 1ml:30mg | China Resources Double-Crane Pharmaceutical Co., Ltd. |
| 3002032ZJ0 | Tanreqing Injection | 10ml | Shanghai Kaibao Pharmaceutical Co., Ltd. |
| 0207013ZS2 | Xiyanping Injection | 2ml | Jiangxi Qingfeng Pharmaceutical Co., Ltd. |
| 1004001ZS0 | Compound Kushen Injection | 5ml | Shanxi Zhendong Pharmaceutical Co., Ltd. |
| 1503074ZS1 | Danshen Polysaccharides Acid Salt Injection | 50mg | Shanghai Green Valley Pharmaceutical Co., Ltd. |
| 0401031ZS1 | Dezocine Injection (Narcotics II) | 5mg:1ml | Yangtze River Pharmaceutical Group Co., Ltd. |
| 0102119ZJ1 | Cefoperazone Sodium and Sulbactam Sodium for Injection | 1.5g (1.0g:0.5g) | Pfizer (China) Pharmaceuticals Co., Ltd. |
| 0102121ZJ1 | Cefotaxime Sodium for Injection | 1g | Hunan Kelun Pharmaceutical Co., Ltd. |
| 0101051ZJ1 | Piperacillin Sodium and Tazobactam Sodium for Injection | 4.5g | Zhuhai Federal Pharmaceutical Co., Ltd. Zhongshan Branch |
| 0101053ZJ1 | Piperacillin Sodium and Tazobactam Sodium for Injection 1.25g | 1.25g | Zhuhai Federal Pharmaceutical Co., Ltd. Zhongshan Branch |
| 0202046ZJ1 | Levofloxacin Hydrochloride Injection | 5ml:0.5g | Yangtze River Pharmaceutical Group Co., Ltd. |
| 1299001ZS0 | Xingnaojing Injection | 10ml | Dali Pharmaceutical Co., Ltd. |
| 1299002ZS1 | Xingnaojing Injection | 5ml | Wuxi JieMinKeXin ShanHe Pharmaceutical Co., Ltd. |
| 3004035ZS1 | Xuesaitong Injection | 400mg | Kunming Pharmaceutical Co., Ltd. |
| 3004110ZS1 | Xuesaitong Powder for Injection | 200mg | Heilongjiang Zhenbaodao Pharmaceutical Co., Ltd. |
| 0699003ZS2 | Shenfu Injection | 50ml | China Resources Sanjiu (Ya'an) Pharmaceutical Co., Ltd. |
| 0699002ZS1 | Shenfu Injection | 10ml | China Resources Sanjiu (Ya'an) Pharmaceutical Co., Ltd. |
| 1506022ZJ1 | Papaverine Hydrochloride for Injection | 30mg | Shandong University High-tech Huatai Pharmaceutical Co., Ltd. |
| 1299006ZS3 | Acetylated Glutamine Injection | 2ml:0.1g | Kangpu Pharmaceutical Co., Ltd. |
| 1503107ZS3 | Shuxuening Injection | 10ml | Langzhi Wanrong Group Co., Ltd. |
| 1004009ZS0 | Kudiezi Injection | 10ml | Shenyang Shuangding Pharmaceutical Co., Ltd. |
| 0112004ZS1 | Meropenem for Injection (0.5g) | 0.5g | Sumitomo Dainippon Pharma Co., Ltd. Suzuka Plant |
| 1699020ZS1 | Shenkang Injection | 20ml | Xi'an Century Shengkang Pharmaceutical Co., Ltd. |
| 1503075ZS1 | Shenxiong Glucose Injection | 20mg:100ml | Guizhou Jingfeng Pharmaceutical Co., Ltd. |
| 1599018ZJ1 | Ginkgo Leaf Extract Injection | 5ml:17.5mg | Yuekang Pharmaceutical Group Co., Ltd. |
| 1401064ZJ1 | Pantoprazole Sodium for Injection | 40mg | Hangzhou Zhongmei Huadong Pharmaceutical Co., Ltd. |
| 1401063PJ1 | Pantoprazole Sodium Enteric-Coated Tablets | 40mg*30 tablets | Aurobindo Pharma Limited |
| 1401065JN1 | Esomeprazole Magnesium Enteric-Coated Capsules | 40mg*30 capsules | Zhengda Tianqing Pharmaceutical Group Co., Ltd. |
| 1401004ZS0 | Omeprazole Sodium for Injection | 40mg | AstraZeneca Pharmaceutical Co., Ltd. |
| 1401071ZJ1 | Omeprazole Sodium for Injection | 40mg | North China Pharmaceutical Co., Ltd. |
| 1401066ZJ1 | Lansoprazole for Injection | 30mg | Jiangsu Aosaikang Pharmaceutical Co., Ltd. |
| 1401051PJ2 | Lansoprazole Enteric-Coated Tablets (28) | 15mg*28 tablets | Yangtze River Pharmaceutical Group Sichuan HaiRong Pharmaceutical Co., Ltd. |
| 1401070PJ1 | Rabeprazole Sodium Enteric-Coated Tablets | 10mg*7 tablets | Eisai (China) Pharmaceutical Co., Ltd. |
| 1401048ZS1 | Rabeprazole Sodium for Injection | 20mg | Jiangsu Aosaikang Pharmaceutical Co., Ltd. |
| 0402019ZS2 | Ketorolac Tromethamine Injection | 1ml:30mg | Shandong New Era Pharmaceutical Co., Ltd. |
| 1401068ZJ1 | Famotidine for Injection | 20mg | Jincheng Haishi Pharmaceutical Co., Ltd. |
| 3004117WJ1 | Mai Luoshutong Pills | 12g*6 bottles | Lunan Houpu Pharmaceutical Co., Ltd. |
| 0202045ZJ1 | Levofloxacin Hydrochloride and Sodium Chloride Injection | 100ml:0.5g:0.9g | Guangzhou Green Cross Pharmaceutical Co., Ltd. |
| 0112002ZS1 | Meropenem Powder for Injection | 0.5g | Shenzhen Haibin Pharmaceutical Co., Ltd. |
| 2107004ZS0 | Human Albumin Injection L | 20%:10g*50ml | Chengdu Rongsheng Pharmaceutical Co., Ltd. |
| 1209041ZJ1 | Edaravone and Sodium Chloride Injection | 100ml:30mg | Jiangsu Chia Tai Fenghai Pharmaceutical Co., Ltd. |
| 1209047ZJ1 | Edaravone Injection | 20ml:30mg | Qilu Pharmaceutical Co., Ltd. |
| 1506019PJ2 | Betahistine Mesylate Tablets (100) | 6mg*100 tablets | Eisai (China) Pharmaceutical Co., Ltd. |
| 1506024PJ1 | Betahistine Hydrochloride Tablets | 4mg*20 tablets | LeP Hengjiuyuan Pharmaceutical Co., Ltd. |
| 1506021ZS0 | Betahistine Hydrochloride Injection | 5ml:30mg | Shijiazhuang Siyao Co., Ltd. |
| 0607024ZJ1 | Compound Amino Acid Injection (18AA-V-SF) | 250ml:8.06g12.5g | Hubei Yibantian Pharmaceutical Co., Ltd. |
| 0607017ZS3 | Compound Amino Acid Injection (18AA) | 30g:250ml | Baifeng Pharmaceutical Chemical Industry Yichang Co., Ltd. |
| 0607018ZS2 | Compound Amino Acid Injection 18AA_V | 250ml | Kangchen Pharmaceutical (Inner Mongolia) Co., Ltd. |
| 0607019ZS2 | Hexa-Amino Acid Injection | 250ml | Shaanxi Jinyu Pharmaceutical Co., Ltd. |
| 0607007ZS4 | Pediatric Compound Amino Acid Injection (Child) | 20ml | China Resources Double-Crane Pharmaceutical Co., Ltd. |
| 0607021ZS5 | Pediatric Compound Amino Acid Injection (19AA-I) (Child) | 6g:100ml | Shandong Qidu Pharmaceutical Co., Ltd. |
